# Non-tropical endomyocardial fibrosis by cardiovascular magnetic resonance, demonstrating the value of CMR in restrictive cardiomyopathy

**DOI:** 10.1186/1532-429X-13-S1-P281

**Published:** 2011-02-02

**Authors:** Christian Hamilton-Craig, Nicholas J Brett, Jodi Harker, Wendy E Strugnell, Richard E Slaughter

**Affiliations:** 1 Centre of Excellence in Cardiovascular MRI, Brisbane, Australia

## Introduction

Non-tropical non-hypereosinophilic left ventricular endomyocardial fibrosis (EMF) is a rare condition, not associated with defined substrates, about which there is a paucity of data in the literature. We describe a series of EMF patients presenting to our institution over 4 years in a high-volume CMR service.

## Purpose

Retrospective review of 4156 clinical CMR studies (May 2006 to August 2010) was undertaken to identify cases of undifferentiated restrictive cardiomyopathy, confirmed on echocardiography and not otherwise meeting current classification guidelines. CMR had been ordered to further elucidate the etiology of restrictive cardiomyopathy. Obtained metrics were compared to a cohort of age-matched controls.

## Methods

CMR was performed on 1.5T system, and dual-read by a cardiologist and radiologist with SCMR level 3 experience. LV volumes, EF, segmental wall thickness, and late gadolinium enhancement were quantified with MASS software. Cases consistent with endomyocardial fibrosis (EMF) were identified and compared to age-matched controls. No patients had history of hypereosinophilia and none were from endemic areas in subtropical Africa.

## Results

**Figure 1:**
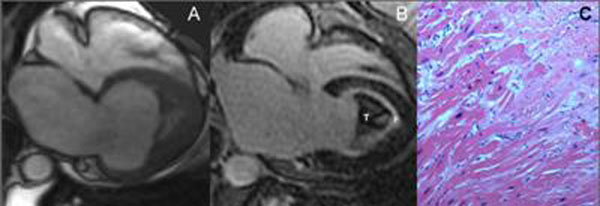
Left Ventricular Endomyocardial Fibrosis A) 4-chamber SSFP, B) LGE showing thrombus (T) and fibrosis (F), C) histologic confirmation.

5 cases (0.12%) of undifferentiated restrictive cardiomyopathy with distinctive features of EMF were identified. ECG was non-diagnostic in all cases, and echocardiography thought to represent the apical form of HCM. Mean age 43 years (range 16-60), 40% female. Mean EF 50% (range 39-59%), controls 61%±2.1(p=0.020). Indexed LV mass was significantly increased (65.6 ±2.6 g/m^2^ vs 46 ±12 g/m^2^ , p=0.012), LV length was significantly shorter (62.2 ±13.2 mm vs 93.5 ±5.1 mm, p =0.0005), and LV apical wall thickness was significantly increased (17 ± 6.6 mm vs 2.5 ± 0.5 mm, p = 0.003). All cases of EMF demonstrated extensive, high-signal subendocardial enhancement in the left ventricle on LGE images. Apical thrombus was present in all cases, but only seen on echo in two. Two patients underwent surgery with histological confirmation of CMR findings. Figure 1

## Conclusions

Non-tropical EMF is distinguished by contracted left ventricular length, increased apical wall thickness, abnormally intense subendocardial fibrosis and presence of apical thrombus. The ventricular architecture and pattern of enhancement differed from the major alternative diagnoses, such as apical hypertrophic cardiomyopathy, left ventricular noncompation and infiltrative myocardial disorders. CMR adds value in the diagnosis of restrictive cardiomyopathies, over-and-above ECG and echocardiography, and can be recommended in cases where ECG and echo data are discordant.

